# Baden Prevention and Reduction of Incidence of Postoperative Delirium Trial (PRIDe): a phase IV multicenter, randomized, placebo-controlled, double-blind clinical trial of ketamine versus haloperidol for prevention of postoperative delirium

**DOI:** 10.1186/s13063-018-2498-6

**Published:** 2018-02-26

**Authors:** Harriet Riegger, Alexa Hollinger, Burkhardt Seifert, Katharina Toft, Andrea Blum, Tatjana Zehnder, Martin Siegemund

**Affiliations:** 1 Department for Anesthesia, Surgical Intensive Care, Prehospital Emergency Medicine and Pain Therapy, University Hospital Basel, Basel, Switzerland; 2 Epidemiology, Biostatistics and Prevention Institute, University of Zurich, Zurich, Switzerland; 3 Department for Anesthesia, Intensive Care and Emergency Medicine, See-Spital, Horgen and Kilchberg branches, Horgen and Kilchberg, Switzerland

**Keywords:** Postoperative delirium, Delirium prevention, Haloperidol, Ketamine, Randomised clinical trial, NSE, S100β, MMSE, Nu-DESC, ICDSC, DOS

## Abstract

**Background:**

Delirium is a neurobehavioural syndrome that frequently develops in the postoperative setting. The incidence of elderly patients who develop delirium during hospital stay ranges from 10-80%. Delirium was first described more than half a century ago in the cardiac surgery population, where it was already discovered as a state that might be accompanied by serious complications such as prolonged ICU and hospital stay, reduced quality of life and increased mortality. Furthermore, the duration of delirium is associated with worse long-term cognitive function in the general ICU population. This long-term experience with delirium suggests a high socioeconomic burden and has been a focus of many studies. Due to the multifactorial origin of delirium, we have several but no incontestable options for prevention and symptomatic treatment. Overall, delirium represents a high burden not only for patient and family members, but also for the medical care team that aims to prevent postoperative delirium to avoid serious consequences associated with it.

The purpose of this study is to determine whether postoperative delirium can be prevented by the combination of established preventive agents. In addition, measured levels of pre- and postoperative cortisol, neuron specific enolase (NSE) and S-100β will be used to investigate dynamics of these parameters in delirious and non-delirious patients after surgery.

**Methods/design:**

The Baden PRIDe Trial is an investigator-initiated, phase IV, two-centre, randomised, placebo-controlled, double-blind clinical trial for the prevention of delirium with haloperidol, ketamine, and the combination of both vs. placebo in 200 patients scheduled for surgery. We would like to investigate superiority of one of the three treatment arms (i.e., haloperidol, ketamine, combined treatment) to placebo.

**Discussion:**

There is limited but promising evidence that haloperidol and ketamine can be used to prevent delirium. Clinical care for patients might improve as the results of this study may lead to better algorithms for the prevention of delirium.

**Trial registration:**

ClinicalTrials.gov, NCT02433041. Registered on 7 April 2015.

Swiss National Clinical Trial Portal, SNCTP000001628. Registered on 9 December 2015.

**Electronic supplementary material:**

The online version of this article (10.1186/s13063-018-2498-6) contains supplementary material, which is available to authorized users.

## Strengths and limitations of the study

- The study’s main strength is the implementation of a promising preventative method for a tenacious problem: the lack of adequate and dependable prevention of postoperative delirium, a condition first described more than five decades ago [1, 2].
- The trial tests the preventative properties of ketamine and haloperidol, which can be demonstrated in a broad field of surgical procedures.
- No adverse events are to be expected from the administration of the trial medication as a weight-dependent single dose following careful consideration of exclusion criteria.
- Evidence from previous studies suggests a considerable benefit for prevention of delirium along with increased comfort and safety for patients involved in the study.
- The Baden PRIDe Trial will primarily recruit patients from two Swiss anaesthesiology departments to achieve the calculated sample size within the foreseen time period.
- The study is limited by the heterogeneous risk constellation of our patients considering the risk factors for delirium that have been gathered to date.
- These variable conditions will not be addressed by the evaluation of a score to assess patient comorbidity (e.g., Simplified Acute Physiology Score II).

## Background

First described more than half a century ago [1], numerous risk factors for delirium have been detected over the last decades [3], emphasising the importance of delirium prevention. Despite of being focus of long lasting research, [4–9] incidence remains high [10, 11]. While study data offer some possibilities, there are no strictly defined rules of action for pharmacological or neuropsychological prevention for delirium in general [12]. Hospitals have various algorithms for prevention and treatment of delirium. This randomised, double-blind, placebo-controlled study aims to compare haloperidol [13] and ketamine [13, 14], two agents that have been suggested to prevent postoperative delirium, separately and in combination.

As with all pharmacological treatment options, drug side effects have always to be taken into consideration and careful observation of patients is mandatory. Common side effects of haloperidol include agitation, sleep disorders, extrapyramidal symptoms, overshooting body and extremity movements and headache. Frequent side effects of ketamine include psychiatric disorders (wake-up reactions such as hallucinations, vivid dreams, nightmares, confusion, motor restlessness, conspicuous behaviour and agitation), neurological disorders (nystagmus, tonic and clonic movements, increased muscle tone and elevated intracranial pressure except for controlled ventilation), eye disorders (diplopia, visual disturbance), cardiac problems (tachycardia, hypertension), respiratory problems (elevated respiratory rate), gastrointestinal symptoms (nausea and vomiting) and cutaneous symptoms (erythema, morbilliform exanthema).

While haloperidol seems to be the drug most often administered for delirium prevention [15] and treatment [12], ketamine demonstrates anti-inflammatory properties that might explain its beneficial effect on limiting delirium emergence [16]. A recently published study found that the administration of a sub-anaesthetic dose of ketamine in patients aged 60 years or older undergoing major surgery did not reduce the incidence of postoperative delirium or affect postoperative pain [17]. In this study, the findings were contrary to the trial hypothesis and conflicted with previously published evidence and guidelines [8, 10, 18].

## Methods

### Study setting

**Fig. 1:**
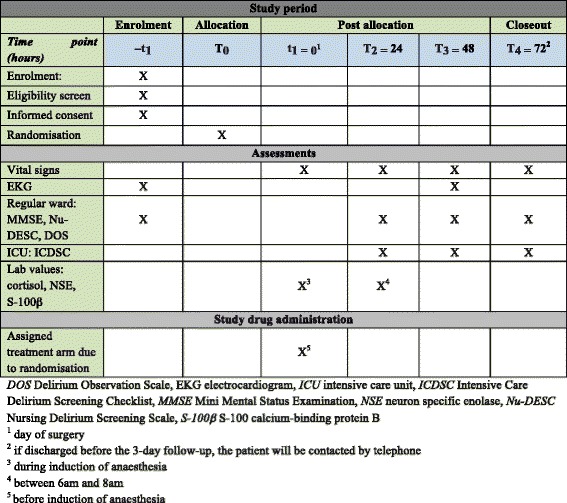
Study period overview (SPIRIT figure)

Patients are recruited in the anaesthesiology departments of the Cantonal Hospital of Baden and the University Hospital of Basel in the preoperative setting. We adhered to the standard protocol items: recommendation for interventional trials (SPIRIT) figure in preparing the schedule of enrolment, interventions and assessments (see Fig. 1) and the SPIRIT checklist for recommendations for standard protocol items for interventional trials (see Additional file 1).

### Eligibility criteria

#### Inclusion criteria

Patients undergoing either elective or emergency surgery can be screened for eligibility. Participants fulfilling the following inclusion criteria are eligible for the study:

- Adult patients age 65 years or older
- Scheduled for surgery within the following fields:
  - ○ Visceral
  - ○ Orthopaedic
  - ○ Vascular
  - ○ Gynaecological
  - ○ Cardiac
  - ○ Thoracic
- Receive general or combined anaesthesia for their surgery
- Signed informed consent agreement

#### Exclusion criteria

Participants meeting the following criteria are excluded from the study:

- Delirium upon hospital admission or during the course of the hospital stay or Mini Mental State Examination (MMSE) score < 24 points or a Delirium Observation Screening Scale (DOS) score ≥ 3 points
- Dementia
- High risk of postoperative treatment in the intensive care unit (ICU) (standard procedure excluded)
- Known haloperidol or ketamine intolerance
- Lack of cooperation or lack of communication possibilities
  - ○ Speech disorders
  - ○ Isolation
  - ○ Aphasia
  - ○ Coma
  - ○ Terminal illness
  - ○ Drug or alcohol abuse
- QT interval (QTc) prolongation (≥ 460 ms in men, ≥ 470 ms in women) or drugs influencing QT interval (see Appendix)
- Parkinson’s disease
- Parkinsonism
- Intake of dopaminergic drugs (levodopa, dopamine agonists)
- Epilepsy
- Delay of surgery for > 72 h after set indication for surgery
- Body weight > 100 kg
- Patient is unable to read German

### Interventions

Participating patients are randomly allocated to one of four study groups: one receiving haloperidol, one receiving ketamine, one receiving both haloperidol and ketamine, and one receiving placebo (Table 1). Patients are administered the active comparator or the placebo comparator only once right before the induction of anaesthesia. Three follow-up days are scheduled beginning on the first postoperative day.

**Table 1:** Overview of treatment arms

Treatment arms	Assigned interventions
Active comparator: haloperidol	Haloperidol 0.005 mg/kg body weight at induction of anaesthesia
Active comparator: ketamine	Ketamine 1 mg/kg body weight at induction of anaesthesia
Active comparators: haloperidol + ketamine in combination	Haloperidol 0.005 mg/kg body weight + ketamine 1 mg/kg body weight at induction of anaesthesia
Placebo comparator	Normal saline (NaCl 0.9%)

The study period consists of enrolment, allocation, post-allocation and closeout (Table 2). Allocation is defined as the time point at which the patient is randomised to one of the four treatment arms. Closeout is defined by either the last day of in-hospital follow up or the follow up assessed by a telephone call in the case of preterm hospital discharge.

**Table 2:** Weight-dependent dosing

Haloperidol: 5 μg/kg body weight diluted in NaCl 0.9%, haloperidol 5 mg/ml concentrate					
Weight (kg)	Haloperidol (mg)	NaCl (ml)	Weight (kg)	Haloperidol (mg)	NaCl (ml)
40–50	0.5	19.5	71–80	0.8	19.2
51–60	0.6	19.4	81–90	0.9	19.1
61–70	0.7	19.3	91–100	1.0	19.0
Ketamine: 1 mg/kg body weight diluted in NaCl 0.9%, ketamine 50 mg/ml concentrate					
Weight (kg)	Ketamine (mg)	NaCl (ml)	Weight (kg)	Ketamine (mg)	NaCl (ml)
40–50	50	19.0	71–80	80	18.4
51–60	60	18.8	81–90	90	18.2
61–70	70	18.6	91–100	100	18.0

The investigators will preoperatively and postoperatively test cognitive function with the Mini Mental Status Examination (MMSE), the Delirium Observation Scale (DOS), the Nursing Delirium Screening Scale (Nu-DESC) or the Intensive Care Delirium Screening Checklist (ICDSC) (Fig. 1). In addition, preoperative and postoperative cortisol, neuron specific enolase (NSE) and S-100 calcium-binding protein B (S-100B or S-100β) levels will be measured. If the study participant is discharged from the hospital before the scheduled 3 follow-up days are terminated he or she will be contacted by telephone and the conversation will be documented in a free-text field on the case report form.

### Outcomes

#### Primary outcome measure

Superiority of one of the active comparator treatment arms in patients after surgery for prevention of hypoactive, hyperactive or mixed delirium, which is assessed by 2-point drop in MMSE score when assessed during one of the 3 postoperative days.

#### Secondary outcome measures

Incidence of postoperative delirium based on cognitive testing and laboratory parameters:

- A 1-point drop in Nu-DESC score on 1 of the 3 postoperative days
- Significant elevation of study-specific laboratory values after surgery:
  - ○ Elevation of NSE above 16.3 μg/l (normal value)
  - ○ Elevation of S-100β above 0.1 μg/l (normal value) or doubling of the preoperative value
  - ○ Elevation of cortisol level above 638 nmol/l (upper limit of normal range)

### Sample size

Sample size was estimated to be able to show the superiority of one treatment arm to placebo by a 30% reduction in the incidence of delirium (efficacy). To achieve power of 0.8, 188 study participants will have to be recruited. To accommodate for drop-out cases the sample size has been increased to 200 (i.e., 50 study participants to be recruited for each treatment arm).

### Recruitment

All consecutive patients aged 65 years or older scheduled for surgery will be screened for study eligibility and then recruited if consent is given.

### Allocation

#### Sequence generation

The allocation sequence follows computer-generated random numbers provided by the Clinical Trials Center (CTC) Zurich. To reduce predictability of the random sequence, the lists are stored in the office of the head nurse with no access permitted by any member of the study team.

#### Allocation concealment mechanism

Study envelopes containing information on the treatment arm allocated to a specific study participant are assembled by designees who are not member of the study team. For each study participant, two sealed envelopes are prepared: one regular envelope to be handed out to the nurses who prepare the study drug and one emergency envelope to be opened in case of an adverse event. The sealed emergency envelope is stored with the documents detailing the patient’s current and past medical history to guarantee complete access in case of an emergency.

#### Implementation

The allocation sequence follows successive numbers from 1 to 200. Participants may be enrolled by every study team member based on the previously described schedule. After consent is given, the sealed envelope that contains the corresponding allocation number is then handed over to the nurse who prepares the study drug. Preparation study drug(s) is controlled by a member of the study team who is not involved in the recruitment process or the follow-up of the specific study participant. The study drug is then administered by the anaesthesiologist who also is not member of the study team. All scheduled delirium assessments, except for the MMSE, are performed by the nurses responsible for the care of the study participant except. The MMSE is conducted by a member of the study team.

### Blinding

Trial participants and care providers including the anaesthesiologist administering the study drug are blinded. The study drugs are prepared as follows to avoid assumption by any colour difference from the placebo (see Table 2 for weight dependent dosing). This adds to a total of 40 ml solution administered slowly to every patient:

1. Trial arm haloperidol: one 20 ml syringe containing the appropriate concentration of haloperidol, and one syringe containing 20 ml NaCl 0.9%
2. Trial arm ketamine: one 20 ml syringe containing the appropriate concentration ketamine, and one 20 ml syringe containing NaCl 0.9%
3. Trial arm haloperidol + ketamine: one 20 ml syringe containing the appropriate concentration haloperidol, and one 20 ml syringe containing the appropriate concentration ketamine
4. Trial arm placebo: two syringes, each containing 20 ml NaCl 0.9%

### Data collection

All participants in the study will be provided a participant information sheet describing the study and providing sufficient information to enable the participant to make an informed decision about their participation in the study. The patient information sheet and the consent form will be submitted to the competent ethics committee to be reviewed and approved. The formal consent of a participant, using the approved consent form, must be obtained before the participant is subjected to any study procedure. The participant should read and consider the statement before signing and dating the informed consent form and should be given a copy of the signed document. The consent form must also be signed and dated by the investigator (or his designee). The signed form will be retained as part of the study records.

The patient will be informed on the possibility to withdraw their data from the study at any time and without needing to provide any explanation. In the case of discontinuation from trial participation, the data collected (i.e., measured laboratory values) will be used for publication if the patient does not object. In the case of deviation from the study protocol, patient data will be destroyed and thus not used for publication. Patients discharged from the hospital before the 3-day follow up post-surgery will be contacted by phone to assess their wellbeing. The collected data will be used for publication in these cases.

Data will be entered into a web-based electronic case report form (eCRF) established by the CTC Zurich (secuTrial®). Paper case report forms will be used in parallel also because of possible technical difficulties. Trial staff will have exclusive access 24 h per day, 7 days per week, to the electronic case report for data entry. A unique patient identification code will be assigned electronically to every randomised patient.

#### Trial medication

Patients enrolled in the trial will be randomised to receive either haloperidol (Haldol®, concentrated 5 mg/ml for intravenous (iv) administration, Janssen-Cilag AG, Zug, Switzerland), ketamine (Ketalar®, concentrated 50 mg/ml for iv administration, Pfizer AG, Zurich, Switzerland), both drugs combined or NaCl 0.9% (NaCl 0.9% B. Braun® for iv administration, B. Braun Medical AG, Sempach, Switzerland) administered right before induction of anaesthesia to prevent postoperative delirium.

#### Delirium assessment tools

For delirium assessment, the MMSE, DOS and Nu-DESC will be assessed preoperatively to avoid inclusion of patients suffering from cognitive decline prior to surgery. These three tests will be assessed daily one first 3 postoperative days to document the course after surgery and to detect the potential development of postoperative delirium. The MMSE and Nu-DESC will be assessed once daily, and the DOS will be assessed during every nursing shift. Data collection forms are available online in German and English.

The MMSE score is an assessment tool used in nearly all medical fields. The MMSE is a 30-item checklist of symptoms suggestive of cognitive decline. Patients lose 1 point for each symptom that manifests during the specified time frame: 1 point is taken if the question cannot be answered, if it is answered incorrectly or if the demanded task cannot be executed by the patient. Overall, orientation in time and place, retentiveness, short-term memory, language and text comprehension, presence of agraphia, apraxia, agnosia, and executive functions (e.g., action planning) are evaluated. A total of 30 points can be achieved. No cognitive decline is suspected in patients who achieve 27 points. A total of 26 points is considered the threshold value. A total MMSE score of fewer than 24 points is indicative of cognitive limitations. Therefore, patients with an MMSE score below 24 points or a DOS score of at least 3 points preoperatively are not recruited for the study.

The DOS represents a screening tool that has been specifically developed for the assessment of conspicuous behavioural traits suggestive of cognitive decline by the nursing team responsible for the care of a patient. It is based on observations made according to a 13-item checklist that is used during every shift, usually over a period of 3 days. In our study, the DOS score is assessed preoperatively and on postoperative days 1–3. One condition for DOS score interpretation is the assessment of an additional tool for the estimation of cognitive function when the DOS score suggests cognitive decline (i.e., DOS score ≥ 3 points) [19].

The Nu-DESC is a delirium screening tool that has been translated into German in accordance with the guidelines [20]. Based on the Nu-DESC assessment tool, Gaudreau et al. have developed an easy-to-use, nursing-based measurement tool that can be easily integrated in everyday practice [20].

If the patient has to be treated in the ICU after surgery, the ICDSC score is documented during every shift. The ICDSC is among the most well-studied and widely implemented adult ICU delirium screening tools worldwide and has been recommended by recently updated clinical practice guidelines. The ICUs of the University Hospital of Basel routinely use the ICDSC for assessment of delirium. Based on high-quality evidence, it has been recommended for the screening of delirium in ICU by the Society of Critical Care Medicine [7]. A score ≥ 4 indicates a positive ICDSC and the presence of delirium [7, 21].

#### Laboratory assessments of cognitive function

##### NSE

Elevated NSE can be measured in the blood (half-life of roughly 48 h) or the cerebrospinal fluid (half-life of 6–8 h) and is indicative of various pathological conditions. It serves as a marker of acute neuronal lesions as in anoxia, acute brain injury, or inflammatory processes leading to cell destruction. NSE levels are negligible after damage to extracerebral tissue. NSE is quantified in acute brain injury, subarachnoid haemorrhage, stroke, status epilepticus, dementia, active multiple sclerosis, severe central nervous system (CNS) infection, decompensated hypertension, intracerebral and extracerebral neoplasia, cardiopulmonary bypass during heart surgery, cardiac arrest, and neurological metabolic diseases for course documentation and prognostic evaluation.

Although elevated NSE is associated with above-mentioned conditions, Rasmussen et al. identified a significant decrease in NSE after abdominal surgery in a study of 65 patients, but this did not correlate with cognitive dysfunction [22]. Other studies with larger cohorts also showed no difference in NSE levels in delirious and non-delirious patients after investigation of elderly patients who were acutely admitted after hip fracture [23]. However, other data suggest correlation between elevated NSE and delirium in the ICU population [24]. We conclude that the role of NSE in the diagnosis of delirium and its possibility to evaluate long-term outcome is still unclear and needs further investigation.

##### S-100β

S-100 proteins play a role in the regulation of various cellular processes (e.g., cell cycle progression and differentiation) and can be localised in the cytoplasm and/or nucleus of numerous types of cells [25]. Altered levels have been connected to several diseases including Alzheimer’s disease [26], Down’s syndrome [27], epilepsy [28, 29], amyotrophic lateral sclerosis [30, 31], melanoma [32–34], type I diabetes [35, 36] as well as in several neurological [37] and neoplastic diseases [38].

Study results on S-100β are also inconclusive for delirium. A study interpreted elevated levels of S-100β as either a marker of cerebral damage or as a consequence of delirium or cerebral damage that could lead to delirium [23]. While delirious patients in this study had the highest levels of S-100β, calling for further investigation to elucidate its role in the pathophysiological pathway that leads to delirium [23], another study found no significant difference in S-100β in delirious compared to non-delirious patients but this was specifically in a critically ill cohort [24]. To resolve this uncertainty, we would like to profoundly investigate this marker in postoperative delirium.

##### Cortisol

Serum cortisol levels have been shown to correlate with both the degree of delirium and the risk of developing it, as stress was found to play a major role in delirium and its pathogenesis postoperatively [39]. Serum cortisol has been suggested to be an important marker of risk of postoperative delirium after cardiac surgery in patients with a preoperative diagnosis of major depressive disorder [40].

Serum cortisol levels depend on the patients’ perioperative stress levels. To date, there remains no clear definition of physiological vs. pathological serum cortisol levels following a certain type of surgery. The present study will analyse cognitive decline or present delirium based on the mentioned scores and correlation between serum cortisol levels preoperatively vs. postoperatively.

#### Electrocardiogram

To evaluate the possible consequences of a potential side effect of haloperidol (i.e., prolongation of the QT interval), we will record an ECG preoperatively and 48 h after surgery. One study demonstrated QT prolongation after intravenous administration of haloperidol to be moderately correlated with the dose administered and recommends clinicians to be aware of this correlation [41]. It is recommended that haloperidol should be discontinued if QTc exceeds 500 ms due to elevated risk of arrhythmia (e.g., torsade de pointes) [42]. Since the elimination half-life is estimated to be 20 h, we will repeat the ECG 48 h after surgery to protect patient safety.

### Data management

All data from this study will be kept within the Trial Master File, and only the study team will have access. In case of a patient’s retroactive denial of study participation, the data collected will not be used for publication in the present or in future trials. In such cases, the data will be destroyed.

All study data will be archived in a designated place on the Surgical Intensive Care Unit at the University Hospital of Basel for a minimum of 10 years after study termination or premature termination of the clinical trial. We plan to store the data also within an eCRF.

### Statistical methods

#### Null and alternative hypothesis

The two-sided statistical null hypothesis will test whether the preoperative administration of haloperidol, ketamine or both drugs combined immediately before induction of anaesthesia compared to placebo is unable to prevent the decline of postoperative cognitive function. The alternative hypothesis is that there is an effect of one of the drugs or the combination.

#### Planned analyses and sample size rationale

##### Primary analysis

Differences among the four groups will be analysed by the chi-square test. Post hoc the groups will be compared by pairwise chi-square test including the Bonferroni correction.

We will follow the intention-to-treat principle. Data on all study participants who have received the study drug as part of one of the four treatment arms will be analysed.

When the sample size in each of the four groups is 50, the 0.05-level chi-square test will have 80% power to detect a difference in proportions characterised by variance (V) of proportions:

V = Σ(π_i_- π_0_)^2^/G of 0.0125 and an average proportion of 0.35

Since study participants will be excluded from the primary analysis if there is a significant decline in MMSE score of at least 2 points during at least 1 of the 3 postoperative days or if they are treated in the ICU after surgery. The determined sample size following power analysis will be increased (see above). This might lead to an uneven number of study participants distributed over the four treatment arms after termination of the study, an occurrence that will need to be taken into consideration.

##### Secondary analyses

These consist of the quantification of the incidence of postoperative delirium and measured changes in the assessed laboratory parameters including NSE, S-100β and cortisol, and the lowest Nu-DESC scoring (of at least 1 point) after surgery. The delirium assessment tools will be evaluated for their reliability. It will be compared if the results of the various assessment tools correlate with diagnosis of delirium.

##### Interim analyses

No interim analyses are planned for this study.

##### Deviation(s) from the original statistical analysis plan

Statistical analyses will begin after all study participants have been recruited for the study. If for whatever reason substantial deviations of the analysis, as outlined in this section, are needed, the responsible Ethics Committees will be informed immediately. All deviations from the analysis detailed in the protocol will be listed and rationalised in the final statistical report.

#### Handling of missing data and drop-outs

Data from study participants who have received the study drug as part of one of the four treatment arms and who were assessed for delirium as declared and who have not been treated in the ICU following surgery can be integrated in the analysis of the primary endpoint. All missing data will be documented in the CRF and described in detail within the publication. Missing values on the secondary endpoint will not be assigned (complete case analyses).

### Data monitoring

No regular monitoring visits at the investigator’s site are planned by the sponsor. The eCRF will be monitored daily by a member of the study team. The source data/documents will be accessible to monitors, and questions will be answered during possible monitoring. The CTC Zurich will enable monitoring at the study centres. The monitoring follows designated standard operating procedures (SOP).

### Adverse events

Individual participants will be excluded from the study in the case of an adverse event that in the opinion of the sponsor contraindicates study drug administration (emergency setting).

### Serious adverse event (SAE)

An SAE is classified as any medical occurrence that results in death, is life-threatening, requires prolongation of existing hospitalisation, or results in persistent or significant disability/incapacity. The occurrence of SAEs will be assessed based on the bedside visit and study of vital and laboratory parameters and will be recorded daily on the eCRF.

All changes in research activity and unanticipated problems will be reported to the competent Ethics Committee by the sponsor and the principal investigator. An SAE or a serious unexpected adverse drug reaction (SUSAR) must be reported within 7 days maximum if fatal, otherwise within 15 days. An annual safety report will be provided by the sponsor.

#### Serious unexpected adverse drug reaction (SUSAR)

A SUSAR indicates an adverse drug reaction that is of a nature or severity that is not consistent with the applicable product information.

### Auditing

To verify trial conduct with respect to good clinical practice (GCP) guidelines, audits and inspections can be executed by the competent authorities involved (i.e., Swissmedic, indicated ethics committees) at any time. Access to all study-specific documents and the corresponding source data is guaranteed at all times. The sponsor and the principal investigator guarantee and answer for all upcoming questions. All involved persons will treat study participant data with full confidentiality.

## Ethics and dissemination

### Research ethics approval

Approval to conduct this study was granted by the Ethics Committee of the canton of Aargau (KEK: 2012–037), the Ethics Committee of Northwestern and Central Switzerland (EKNZ: AGSO 2012/037) and the Competent Authority (Swissmedic: 2013DR4089). The study is registered at the Swiss National Clinical Trial Portal (SNCPT; Identifier: SNCTP000001628) and at ClinicalTrials.gov (Identifier: NCT02433041).

This study is conducted in compliance with the protocol, the current version of the Declaration of Helsinki, the ICH-GCP or ISO EN 14155 (as far as applicable) as well as all national legal and regulatory requirements.

### Protocol amendments

No amendments have been declared prior to submission of the protocol for publication.

### Consent or assent

The investigators will explain to each participant the nature of the study, its purpose, the procedures involved, the expected duration, the potential risks and benefits and any discomfort it may entail. Each participant will be informed that the participation in the study is voluntary, that he/she may withdraw from the study at any time and that withdrawal of consent will not affect his/her subsequent medical assistance and treatment. The participant must be informed that his/her medical record may be examined by authorised individuals other than their treating physician. Only members of the study team will be eligible to seek patient consent or assent for study participation.

### Confidentiality

Collection, documentation, saving and interpretation of personal data in conjunction with this clinical trial conforms to updated Swiss data protection regulations. Voluntary participation of the study participant granted by his or her signature in the study-specific consent form is a prior condition of this study.

Data collected from the study participants during this trial are treated as highly confidential and cannot be transferred to third parties. Confidentiality is guaranteed by assignment of a participant ID number without the possibility of inferring the participant’s identity. With prior consent of the study participant, his or her information can be forwarded to the family doctor or other treating physicians to assure the patient’s well-being. The collected study-specific data can be inspected and reviewed for verification purposes by monitors of the involved ethics committees or other competent authorities.

### Declaration of interests

Each of the authors declares that there are no conflicts of interest.

### Access to data

See Data management.

### Ancillary and post-trial care

Apart from the 3 days scheduled for follow up after surgery, no additional follow up is planned for this study. In the case of any questions or concerns after hospital discharge, contact data are indicated within the patient information form handed out to gain the patient’s consent for study participation.

To compensate those who suffer harm from trial participation, insurance will be provided by the sponsor based on the liability insurance of the Cantonal Hospital of Baden and the University Hospital of Basel.

### Dissemination policy

Study results will be communicated to patients based on expected speed-up in recruiting all 200 foreseen study participants. During the ongoing study and until publication, there will be no public access to the data. We plan to publish the data in a major peer-reviewed clinical journal. A public description of the study in German will be available on the Swiss National Clinical Trial Portal (SNCTP) after gaining approval for conduct of the study from the competent ethics committee.

## Discussion

### Trial rationale

Delirium is a serious condition calling for prompt diagnosis and treatment. We hypothesise that the administration of haloperidol, ketamine or both combined immediately before induction of anaesthesia compared to placebo will lead to a reduced incidence of postoperative delirium. Clinical care for patients might improve as the results of this study may lead to better algorithms for the prevention of delirium. By achieving the goal, this trial could reduce the burden on family members and might protect the patient’s long-term autonomy and health and eventually diminish delirium-associated mortality.

### Population

This study will include patients admitted to the hospital prior to scheduled or emergency surgery without preoperative impaired cognitive function.

### Intervention

There is limited but promising evidence that haloperidol and ketamine can be used to prevent delirium. In our trial, we aim to confirm the superiority of one or more of the non-placebo treatment arms over placebo for the prevention of delirium.

### Outcome

Based on evidence that haloperidol and ketamine both prevent delirium, we chose to evaluate the effect of these drugs, administered alone or in combination, on the incidence of postoperative delirium to be able to calculate a significant reduction and thereby prove our hypothesis.

### Sample size

As described above, sample size was estimated to be able to show the superiority of one of the three non-placebo treatment arms compared to placebo on reducing the incidence of postoperative delirium.

### Perspective

The Baden PRIDe Trial aims to reduce the incidence of postoperative delirium by investigating the three non-placebo treatment arms for the development of a prevention regime for postoperative delirium based on high-quality data.

## Trial status

The ethics committee of the canton of Aargau granted approval of this study in May 2013. Inclusion of first patient was in July 2013. Between July 2013 and August 2017, a total of 171 patients were recruited. The final results are expected in the first half of 2018. Due to ongoing data collection, currently no data are publicly available. Results will be submitted to peer-reviewed journals for publication and will be presented at relevant academic conferences.

### Additional file

Additional file 1: SPIRIT checklist. (DOC 115 kb)

## Appendix

**Table 3:** Overview of drugs causing QT prolongation (Schweiz Med Forum 2007;7:814–819)

Drug group	Drug
Antiarrhythmic agents	Amiodarone (Cordarone ®)
	Disopyramide (Norpace ®)
	Quinidine (Kinidin-Duriles ®)
	Sotalol (Sotalex ®)
	Flecainide (Tambocor ®)
	Ibutilide (Corvert ®)
Other cardiovascular drugs	Dobutamine, dopamine
	Ephedrine
	Epinephrine
	Indapamide (Fludex SR ®)
	Isradipine (Lomir SRO ®)
	Midodrine (Gutron ®)
	Norepinephrine
Psychotropic drugs	Amitriptyline (Saroten ®)
	Chloral hydrate (Chloraldurat ®, Medianox ®, Nervifene)
	Citalopram
	Chlorpromazine (Chlorazin ®)
	Clomipramine (Anafranil ®)
	Doxepine (Sinquan ®)
	Felbamate (Taloxa ®)
	Fluoxetine (Fluanxol ®, Deanxit ®)
	Galantamine (Reminyl ®)
	Haloperidole (Haldol ®)
	Imipramine (Tofranil ®)
	Levomepromazine (Nozinan ®)
	Lithium (Priadel ®, Neurolithium ®)
	Methadone (Ketalgin ®)
	Methylphenidate (Concerta ®, Ritalin ®)
	Nortriptyline (Nortrilen ®)
	Olanzapine (Zyprexa ®)
	Paroxetine (Deroxat ®, Parexat ®)
	Quetiapine (Seroquel ®)
	Risperidone (Risperdal ®)
	Sertindole (Serdolect ®)
	Sertraline (Zoloft ®, Gladem ®)
	Thioridazine (Melleril ®, Melleretten ®)
	Tizadinine (Sirdalud ®/-MR)
	Trimipramine (Surmontil ®, Trimin ®)
	Venlafaxine (Efexor ®)
Gastrointestinal	Dolasetrone (Anzemet ®)
	Domperidone (Motilium ®/-lingual)
	Granisetrone (Kytril ®)
	Octreotide (Sandostatine ®)
	Ondansetrone (Zofran ®)
	Phentermine (Adipex ®)
	Sibutramine (Reductil ® 10/15, Ionamin ®)
Respiratory	Salbutamole (Ventolin ®)
	Salmeterole (Serevent ®, Seretide ®)
	Terbutaline (Bricanyl ®)
Antibiotics	Azithromycine (Zithromax ®)
	Ciprofloxacine (Ciproxin ®)
	Clarithromycine (Klacid ®)
	Erythromycine (Erythrocin ®)
	Levofloxacine (Tavanic ®)
	Moxifloxacine (Avalox ®)
	Ofloxacine (Tarivid ®)
	Roxithromycine (Rulid ®)
	Trimethroprim-Sulfamethoxazole (Bactrim ®, Cotrim ®)
Antiviral agents	Amantadine (Nivaquine ®, Chlorochin ®)
	Mefloquine (Lariam ®, Mephaquin ®)
Antifungal agents	Pentamidine (Pentacarinat ®)
	Fluconazole (Diflucan ®)
	Itraconazole (Sporanox ®)
	Ketoconazole (Nizoral ®)
	Voriconazole (Vfend ®)
Miscellaneous	Alfuzosine (Xatral ®)
	Phenylephrine
	Phenylpropanolamine (Kontexin ®, Rhinotussal ®)
	Pseudoephedrine (Otrinol ®, Benical ®)
	Tacrolimus (Prograf ®, Protopic ®)
	Tamoxifen (Tamoxifen ®, Nolvadex ®)

## Abbreviations

CNS: Central nervous system
CTC: Clinical Trials Center (Zurich)
DOS: Delirium Observation Scale
ECG: Electrocardiogram
eCRF: Electronic case report form
ICDSC: Intensive Care Delirium Screening Checklist
ICH-GCP: International Council for Harmonisation of Technical Requirements for Pharmaceuticals for Human Use - good clinical practice
ICU: Intensive care unit
MMSE: Mini Mental Status Examination
NSE: Neuron specific enolase
Nu-DESC: Nursing Delirium Screening Scale
PRIDe: Prevention and Reduction of Incidence of postoperative Delirium Trial
S-100β: S-100 calcium-binding protein B
SAE: Serious adverse event
SNCTP: Swiss National Clinical Trial Portal
SOP: Standard operating procedure
SUSAR: Serious unexpected adverse drug reaction
